# Microbicide clinical trial adherence: insights for introduction

**DOI:** 10.7448/IAS.16.1.18505

**Published:** 2013-04-08

**Authors:** Cynthia Woodsong, Kathleen MacQueen, K Rivet Amico, Barbara Friedland, Mitzy Gafos, Leila Mansoor, Elizabeth Tolley, Sheena McCormack

**Affiliations:** 1 International Partnership for Microbicides, Silver Spring, MD, USA; 2 FHI 360, Durham, NC, USA; 3 Center for Health Intervention and Prevention, University of Connecticut, Storrs, CT, USA; 4 Population Council, New York NY, USA; 5 MRC Clinical Trials Unit, London, UK; 6 Centre for the AIDS Programme of Research in South Africa (CAPRISA), University of KwaZulu-Natal, Durban, South Africa

**Keywords:** vaginal microbicides, HIV prevention, clinical trial adherence

## Abstract

After two decades of microbicide clinical trials it remains uncertain if vaginally- delivered products will be clearly shown to reduce the risk of HIV infection in women and girls. Furthermore, a microbicide product with demonstrated clinical efficacy must be used correctly and consistently if it is to prevent infection. Information on adherence that can be gleaned from microbicide trials is relevant for future microbicide safety and efficacy trials, pre-licensure implementation trials, Phase IV post-marketing research, and microbicide introduction and delivery. Drawing primarily from data and experience that has emerged from the large-scale microbicide efficacy trials completed to-date, the paper identifies six broad areas of adherence lessons learned: (1) Adherence measurement in clinical trials, (2) Comprehension of use instructions/Instructions for use, (3) Unknown efficacy and its effect on adherence/Messages regarding effectiveness, (4) Partner influence on use, (5) Retention and continuation and (6) Generalizability of trial participants' adherence behavior. Each is discussed, with examples provided from microbicide trials. For each of these adherence topics, recommendations are provided for using trial findings to prepare for future microbicide safety and efficacy trials, Phase IV post-marketing research, and microbicide introduction and delivery programs.

## Introduction

After two decades of clinical trials testing a wide array of products and hoped-for protective mechanisms, the CAPRISA 004 trial provided evidence that a vaginal microbicide containing an antiretroviral (ARV) (tenofovir) could protect against sexual transmission of HIV [1]. It is still an open question whether the findings regarding the effectiveness of coitally associated tenofovir gel use will be confirmed. However, increasing evidence supporting the potential efficacy of vaginally delivered antiretrovirals has stimulated interest in what will be required to most efficiently introduce effective products. Such products will fill an important need for women and girls, who accounted for an estimated 1.2 million of the world's new HIV infections in 2011 and who often have limited ability to negotiate for their personal protection with their partners [2].

A microbicide product with demonstrated clinical efficacy will only be effective if it is used correctly and consistently. Evidence about factors that will likely influence uptake as well as correct and consistent use of vaginal microbicide products proven to be effective is included in the data collected in microbicide clinical trials, as well as ancillary social and behavioural science research. To date, scientific discourse regarding study-product adherence in clinical trials has largely centred on motivating adherence to support the primary objective of the trial and the accuracy of measurement to aid interpretation of results. For the introduction of effective microbicide products into real-world communities (which we refer to here as “microbicide introduction”), the emphasis shifts to measuring uptake, and the interest in adherence is to support individuals to reduce their risk by achieving correct and consistent use. Information on adherence that can be gleaned from microbicide trials is relevant for future microbicide safety and efficacy trials, pre-licensure implementation trials, Phase IV post-marketing research, and microbicide introduction and delivery. This article reviews such information and assesses the implications for microbicide introduction, drawing upon the adherence information primarily from the large safety and efficacy microbicide trials completed to-date. Since topical vaginal products have been the most intensely studied (compared to rectal products), lessons learned from these trials predominate, and our focus is on vaginal microbicide use.

## Background

### Adherence in microbicide trials

Adherence considerations in microbicide trials can be conceptualized in three broad areas: (1) achieving correct and consistent product use, (2) measuring product use and (3) ensuring visit completion and trial retention. Difficulties in measuring adherence in microbicide trials have been widely observed and well-documented [3–7]. Adherence is framed, defined and measured almost exclusively as a modifier of product efficacy [8,9]. The search for an objective measure of adherence as an effect modifier is constrained by the limitations of self-report and biological markers. Self-reported adherence data are prone to error due to participants intentionally or unintentionally misreporting use, staff errors in data collection and lack of clarity about what is being measured. Biological markers are limited by the cross-sectional time-frame for a given measurement, practical constraints on collection of random unannounced specimens, the likelihood of more adherent product use prior to scheduled clinic visits and difficulties of measuring some drugs that are not systemically absorbed [4]. In spite of these challenges, some trials have reported increased efficacy correlated with defined levels of adherence [1,10]. The use of composite measures or triangulated indicators has proven especially useful [11]. For example in the CAPRISA 004 tenofovir gel trial, the relationship between adherence and effectiveness was assessed using a composite measure based on self-reported sexual activity and used applicator counts combined with an assessment of cervico-vaginal fluid drug levels [1].

Counselling approaches adopted in recent HIV prevention trials have shown some promise for improvements in counselling to increase adherence [12]. These have drawn on demonstrated success in achieving ARV treatment (ART) adherence [13–15] and other risk reduction programs. However, little evidence is available regarding the impact of these approaches on the use of products in the context of a microbicide trial, where participants are blinded to whether they are using the active test product or a placebo. Behavioural models for pre-exposure prophylaxis adherence have emerged [12,16,17], but evaluation of the utility of these models is needed. Moreover, since no single adherence counselling approach will work for all contexts, specific incorporation of socio-cultural factors should be part of any explanatory model or proposed intervention approach [17,18]. For example, the gender dynamics and socio-cultural norms influencing behaviour of heterosexual couples in sub-Saharan Africa may preclude the type of adherence negotiation indicated by a model based on United States norms.

A high rate of visit completion and high retention are considered critical components for achieving adherence in microbicide trials; participants who miss visits also miss the opportunity to refill product, which may result in non-adherence or non-persistent use, and ultimately can undermine the accuracy of efficacy estimates. Additionally, those who miss visits or who are lost to follow-up negatively impact the overall ability of the trial to determine product efficacy. However, it is important to note that the very high rates of retention in studies do not appear to ensure high rates of actual product use. Thus, trial retention is a necessary, but insufficient, condition for consistent product use. Further, the ability of trials to retain participants for periods of one to three years may not be indicative of product continuation rates for similar periods of time. Generalizability depends on the extent to which factors driving trial retention are similar to factors that will influence continuation and persistent use of product outside of a trial setting.

## Microbicide trial experiences

A variety of adherence lessons and associated recommendations that arise from these lessons are summarized in Table 1. Table 2 provides a brief overview of the trials from which these lessons have been learned. Below, we discuss each major area, drawing examples from the trials.

**Table 1:** Lessons learned from microbicide trial adherence experiences, and recommendations for future delivery programs, trials and research

Lessons learned from microbicide trials	Recommendations for future delivery programs, trials, operations research and implementation research
**1. Adherence measurement** Self-reported adherence appeared exaggerated in all trials Biological markers of adherence reveal much lower levels of adherence. Triangulation of data offers the opportunity to probe and understand inconsistencies in self-report. Over-reporting may be due to social desirability bias. Bias may be introduced by the way the questions are worded, or the summary measures used for reporting adherence. Over- and under-reporting may be due to forgetfulness or inaccurate recording by staff.	Counselling for correct and consistent use should build on behaviour change best practices to improve adherence reporting in trials and continued use of effective products. Evaluation of counselling will also be needed. Acknowledging that 100% use is unrealistic for some women may improve accuracy of self-report. Triangulation of multiple measures should improve accuracy of adherence measurement. Prescribers and those supporting adherence must be well trained to listen and appropriately encourage accurate reporting of product use. Outcome based measures of adherence (such as HIV infection) will be relevant for microbicide service delivery.
**2. Comprehension of use-instructions** Trials have observed that some women misunderstood instructions on how to correctly use microbicides. The use of locally appropriate illustrated materials has facilitated counselling about correct product use. Participants who are anxious about use generally overcome their concerns. Women may be reluctant to use microbicides during menses.	Social marketing research is needed to develop instructions for users and counselling messages for dispensing staff. These messages should be evaluated. Service provider training will be required for introduction of this new class of product. Initial counselling about use should acknowledge common concerns and provide reassuring evidence-based information on how others have overcome similar concerns. Guidance on use during menses is needed. For continuous-use methods, information is needed on how quickly a protective effect can be achieved after product use is initiated or re-initiated since some women will discontinue or disrupt use during menses.
**3. Unknown efficacy and its effect on use-adherence** Not all participants and staff believe the message of uncertain efficacy, even though they understand it. Participants may believe that a placebo gel “protects” them because the lubrication provided makes sex more comfortable (“safe”). Communication about unknown level of efficacy, targeted efficacy and lack of placebo efficacy has been misinterpreted by some participants and clinic staff.	Messages about unknown efficacy are qualitatively different than messages about partial efficacy, and thus comprehension of partial efficacy must be studied and effective communication strategies developed. It will be important to monitor risk behaviour, and biological markers of risk such as sexually transmitted infections in case there is a decrease in condom use once a product is known to be effective. Users need information on contraceptive effect and effect on male partner since in the absence of such information, incorrect assumptions about protective effects could increase risk of infection and/or unintended pregnancy.
**4. Partner influence on use** Type and nature of sexual relationship is likely to influence use. There is considerable variability in frequency of sexual intercourse amongst participants and over time. User's experiences and expectations for microbicides’ impact on sexual pleasure for themselves and their partner will influence use. Willingness or intention to discuss and/or disclose use is associated with partner type.	Counselling for users should include consideration of partner type and sexual frequency, with counselling messages for use-strategies tailored to these variations. Relationships and associated risk change over time and thus protection needs will change over time. Users’ relationships and needs should be periodically assessed. Messages about microbicide effects on men's and women's sexual pleasure should be crafted in accordance with local norms. Messages about the potential for covert use should include consideration of partner type and the potential for negative consequences if use is discovered.
**5. Retention and continuation** Retention in clinical trials has been high over 1–2 years, but it is unclear what this portends for continuation in non-trial settings when women are not reimbursed, yet know they are receiving an effective product. Retention does not necessarily predict continuation of product use, with evidence suggesting that product use during trials may decrease over time despite re-supply during study visits. Despite requirements for contraceptive use, and counselling to avoid pregnancy, many trial participants have been taken off product due to pregnancies. Women value counselling, HIV testing and other services provided in trial settings. Some participants, partners and communities are concerned about blood testing, including the volume of blood taken, frequency, and dispensation of samples.	Quarterly visits for re-supply appear to be feasible, at least with initial introduction, but should be integrated with other services. Reasons for product discontinuation should be explicitly considered and appropriate counselling developed, including pregnancy, breastfeeding and life circumstances that could result in reduced HIV risk for a woman. Requirements for refills should be streamlined and additional services made available at refill locations. Transportation vouchers or other benefits could incentivize consistent use at early stages of microbicide introduction. Periodic reassessment of women's needs, and consultation about changes in risk should be done to maximize continuation. Women need clear information about when and why to discontinue use (e.g. during pregnancy) and to seek medical advice for continued use. Provision of additional services (at least counselling) may improve willingness to access re-supply services. Access points providing re-supply only (e.g. between quarterly clinic visits) may be sufficient for some users and some products. The type of counselling most effective in a given population requires targeted study. Research is needed to inform the minimal frequency and type of HIV testing that is necessary for ARV-based products, and this minimum should be required for service delivery. Continued community engagement is essential to address and monitor concerns about microbicide products.
**6. Generalizability of trial participants’ adherence behaviour** Women in a range of socio-cultural settings and at high risk of infection find vaginal microbicides to be acceptable and easy to use. Women may not consider themselves to be at risk even in countries where HIV is prevalent. Some important population groups have not been included in clinical trials. Microbicide trials have been conducted primarily in East and Southern Africa, followed by sites in India, United States, West Africa and Thailand.	Studies are needed for populations who have not been well represented in microbicide trials to-date (specifically, adolescent women, pregnant women, older women) to assess potential additional contextual issues that may influence uptake, adherence and continuation. Programs should include counselling to help women assess their risk. This could potentially motivate adherence. Secondary analyses of clinical trial data should be explored, stratified by variables such as age, risk, etc., to determine possible correlates of adherence and continuation of use. Research will be needed in regions of the world where microbicide trials have not concentrated since sexual behaviours and norms likely to influence adherence may differ.

### Adherence measurement in clinical trials

Accurate measurement of product use has been challenging, with most trials observing that microbicide adherence is over-estimated by product count or self-reported measures [10,24]. Understanding the reasons for over-reporting can nonetheless provide valuable insights. Counselling provided in the early microbicide trials emphasized a goal of 100% adherence, which may have contributed to a social desirability bias to over-report. “Person-centred” approaches that focus on what is reasonable and actionable for participants have been adopted in more recent trials and have shown some promise [1,12]. Although social desirability is a key underlying factor, the wording of questions may discourage a negative response and added procedures in response to reports of non-adherence may influence future reporting behaviours. For example, if participants soon learn that reporting non-adherence results in a longer clinic visit, they may be less inclined to report non-adherence at subsequent visits. Several of these concerns have been commonly reported with regard to measuring ART adherence as well, leading to recommendations for short or manageable recall periods, clear and neutral wording of adherence questions and incorporation of a permission statement or acknowledgement that it is not uncommon to miss “doses” on occasion [25,26].

Adherence measurement will be needed for future trials, including Phase IVs, as well as for service delivery indicators. For ARV-based microbicides, drug levels combined with self-report may be necessary tools for assessing adherence during early roll-out. However, over time, less emphasis should be placed on these measures and more on other outcomes (e.g. uptake, retention in the program, HIV breakthrough infections and incidence, HIV resistance) for assessing program success. Intensive or expensive objective measures of adherence will likely become increasingly less feasible and good proxy measures of high adherence predictive of decreases in HIV incidence will be needed. In that context, adherence measures that were too statistically “noisy” for trial purposes may in fact be useful for programmatic monitoring and evaluation purposes. For example, it may be adequate to determine whether adherence exceeds a critical threshold value for effectiveness rather than estimating an exact level of adherence.

### Comprehension of microbicide use-instructions

The products tested in microbicide trials have required either daily/continuous use, or coitally associated use. Microbicide trials tend to provide product use-instructions that include culturally appropriate pictorial diagrams rather than the text-heavy printed instructions that are typical of package inserts in developed-countries. Clinical trial participants are provided with regular education and counselling about use requirements and have access to knowledgeable health professionals to answer questions about use. However, trials have nonetheless observed numerous erroneous beliefs about how to correctly use microbicides. Some trial participants came to their own conclusions about how to correctly use the vaginal products, and these practices could be harmful and/or reduce efficacy. For example, anecdotal information from the HPTN 035 microbicide trial of coital use of PRO 2000 gel suggested that some women erroneously thought that the gel applicator needed to be inserted very high into the vagina, thereby creating the potential for physical damage to the cervix. There were also anecdotal reports that some women thought the gel needed to get washed out of the vagina after sex. In the Carraguard Phase 3 trial, and the MDP301 Pilot, some women misinterpreted the instruction to insert gel up to 1 hour prior to sex to mean that they had to wait an hour after inserting the gel before having sex. Information about the need for condom use along with the microbicide has provided additional confusion and uncertainty about microbicide products. In the absence of correct understanding of the information given, trial participants as well as future users will reach their own conclusions when interpreting use-instructions. This underscores the importance of conducting pilot studies in advance of product introduction, as well as evaluations with early introduction programs, to ensure that the information is presented clearly and correctly understood by intended microbicide users.

A number of microbicide trials have documented the newly enrolled participants’ expectations about microbicides prior to their initial use and how these pre-use expectations compare to use-experience. Trials have generally reported that products were either better than or similar to what was expected and comfort with use appears to increase during the trials. The pre-use expectations documented in microbicide trials may be similar to those of future new “consumers”, as they likely reflect cultural and community norms for sexual behaviour and practices. The expectations and post-use experiences of trial participants could be informative for development of use-counselling messages and package inserts.

Minimal attention has been given to the impact of menstruation on microbicide use-adherence, with scant findings reported in the literature [27]. Recent microbicide trials generally require participants to be using effective contraceptive methods while in the study, and contraceptive-related amenorrhea is quite common. Nevertheless, since most women menstruate at some points in their sexually active lives, the potential menses-related adherence issues must be considered. In sub-Saharan Africa, evidence from multiple cultural contexts suggests that sexual activity tapers during menses [28–30]; thus, in many settings adherence during menses may be a non-issue for coitally dependent methods. For those who have sex during menses, the combination of microbicide gel products and menstrual flow might cause excessive leakage, or make the vagina too lubricated and adherence might drop. The HPTN 059 Phase II study of tenofovir gel, conducted in India and the U.S., reported that menses was given as the main reason for non-adherence [31]. Microbicides requiring continuous use may be discontinued during menses, and not resumed in time for adequate protective drug levels to be reached.

### Unknown efficacy and its effect on use-adherence

The effect of unknown efficacy of microbicide trial products has multiple dimensions. Trial participants are told and regularly reminded that the “active” product may not protect them; that they may have been assigned to use a placebo; and that while the “active” product has unknown protective benefits, it may also have unknown adverse effects. Nevertheless, some trial participants in the HPTN 035 trial (and their partners) believed that both partners were protected by a study product in a placebo-controlled, blinded trial [32]. A proven effective product will certainly be less than 100% effective, and it can be expected that users may over-estimate the protection provided and/or assume a “forgiveness factor” that can accommodate inconsistent use. However, the concept of partial efficacy can be difficult to explain to policy makers, community workers and potential microbicide users. Future users may erroneously calculate what partial efficacy means, and think in terms of additive effects (e.g. if microbicide is 40% effective and male circumcision is 60% effective, microbicide use with a circumcised partner provides 100% protection; or, a woman using a 40% effective microbicide who believes there is a 50–50 chance her partner is HIV+, may believe she has a 90% level of protection).

Future users and those advising on use will need clear information about levels of and requirements for protection. Although to-date, vaginally delivered ARVs have been shown to be effective in protecting only women, not the male partner [1], an erroneous assumption of protection for both partners could contribute to male partner discontinuation of condom use. Women who use ARV-based products will have regular HIV tests confirming that they are not infected. However, infection may occur between HIV tests and partners of women using microbicides may over-estimate the likelihood that their partner must be HIV negative. Furthermore, since the first microbicide products will likely be recommended for the protection of women only, men may be reluctant to support use.

Participants and health professionals may also assume that microbicides have a contraceptive effect, thinking that the product inactivates or “kills” everything associated with an ejaculate. Although health professionals may be unlikely to recommend a woman discontinue use of a highly effective contraceptive method, future microbicide users may reach their own conclusions and inappropriately use a microbicide for dual purposes. A number of products are in development with the goal of providing combined contraception and HIV protection; thus, care must be taken to avoid confusion about the potential array of products. This will be particularly true when introducing an HIV preventive vaginal ring in settings where contraceptive vaginal rings are available. If a microbicide is erroneously assumed to be contraceptive, this could increase adherence, yet contribute to unintended pregnancies.

### Partner influences on use

Evidence from microbicide trials strongly indicates that the decision to use microbicides and the ability to use them correctly and consistently will likely be influenced by the aspects of women's sexual relationships [33–37]. Although microbicides have long been heralded as a potential method for women to use without their partner's active involvement, cooperation or knowledge, the realities of using a microbicide in secrecy or without potential for partners to notice may create a high burden and challenge adherence. Early microbicide trials indicated that women generally told their partners about trial participation for a variety of reasons: some women considered that lengthy trial participation with regular visits would be hard to keep secret, some felt it was wrong to not tell a partner in a committed relationship (e.g. husband) or felt that a decision like this should be made as a couple, some were afraid of the consequences of being discovered, and concerns were expressed about microbicides negatively impacting the partner's sexual pleasure [38,39]. As awareness of these issues accumulated among the microbicide research community, many trials encouraged women to consider telling their partner, because it was felt that partner support could improve adherence and reduce the potential for social harms.

When effective microbicides are introduced, some women will likely achieve higher adherence with partner support, some may be highly motivated to use products discretely, and some may consider that use and disclosure are their own decisions, regardless of the partner's views. The ability to use discretely may vary by product type and use-regimen. Trial evidence indicates that women may be more likely to use microbicides without disclosure to casual or paying partners than with long-term steady partners, and adherence rates have been observed to differ according to partner type [22,40].

### Retention and continuation

Microbicide trials include active outreach, reimbursements and care referrals to help achieve the high retention needed to support a valid result. Continuation of use, rather than “retention”, is the relevant concern for microbicide delivery, and this concept has somewhat different aspects for consideration. Clinical trials have achieved high retention rates (Table 2). However, future users will not be reimbursed for the costs associated with visiting the refill source, and they may not receive the additional services or reminders to return that are currently used in clinical trials. How HIV testing requirements, which have been monthly to-date in the ARV microbicide trials, will impact willingness to continue use will be difficult to predict since some women consider regular testing to be a benefit of microbicide trial participation, while others dislike it because of fears about blood testing, in general, and the potential for a positive result.

**Table 2:** Overview of Microbicide Clinical Trials

Clinical trial	General description	Population description	Method for recoding adherence; adherence rate; trends noticed	Retention rate (%)
Col 1492 N-9; Phase III [19] a,e,f	Vaginal gel (pre-coital); n=765; 48 weeks; 1-monthly visits	Age: 16+; 100% Sex workers	Self-report: difference by partner type, high self-reported use	68
HPTN 035; Phase IIb [20] a,b,c,d	Vaginal gel (pre-coital), n=3,099; 12–30 months (av. 20); 1-monthly visits	Age: 18–56 (but few above age 45); 62% married	Self-report: 81% last sex acts	94
Population Council Carraguard; Phase III [21] a	Vaginal gel (pre-coital); n=6,202; 9–24 months; 3-monthly visits	Age: 16+; 63% single/never married; 31% married/living as married	Self-report: 96% of women reported using gel at last sex act; applicator dye test: gel used in 42% of sex acts, overall	69
CONRAD Cellulose Sulfate; Phase III [22] a,b,c,e	Vaginal gel (pre-coital); n=1,398; 12 months; 1-monthly visits	Age: 18+; 23% married; 3 partners previous 3 months	Self-report: 87% overall, 46% use when condoms not; lower use with primary partners	Study stopped early
MDP 301 PRO2000/5; Phase III [23] a,b	Vaginal gel (pre-coital); n=9,385; 12 months (24 in Uganda); 1 monthly visits	Age: 16+; Sexually active but less than 14 acts per week; 99% in stable relationships	Self-report and used/unused applicator count: 89% last sex act; 56% last sex act at every visit	88
CAPRISA 004 Tenofovir; Phase IIb [1] a	Vaginal gel (pre- and post-coital); n=889; 30 months; 1-monthly visits	Age: 18–40; mean 23.9;±5.1; Sexually active with at least 2 vaginal sex events in previous 30 days; 95% with one stable partner in previous year; 12.3% living with regular partner; 2% ever received money in exchange for sex	Self-report and used and unused applicator return (sub-samples: method developed to confirm if used applicator returned was in fact used; “wise-bag” to determine when applicator removed); 18-month decrease in efficacy may be due to adherence drop due to trial fatigue	95

Trial sites:

a Southern Africa

b East Africa

c India

d United States

e West Africa

f Thailand.

Once the reimbursement, transport support and outreach efforts of clinical trials are removed, women may have difficulty accessing microbicides to maintain correct and consistent use. Microbicides will be a new class of product, and it is not clear as to the service delivery setting in which they will be provided. The type of access needed will vary by the type of product and re-supply schedule. Current products under evaluation range from a single vaginal ring used continuously for at least a month at a time, to boxes containing a dozen or more pre-filled gel applicators for coitally associated use. Depending on where service delivery is situated, service providers may be overwhelmed and confused by yet another service to deliver, and they will likely have fewer resources and less time to spend with women than what was provided in trials. As with use of other health services, continued use will likely be influenced by the way women are treated by providers. There are likely to be significant differences between the clinical research environment and typical health service settings, ranging from amenities (e.g. refreshments, available seating) to staffing (e.g. ratio of staff to clients, workload, salary) to time spent at the facility (e.g. waiting in general waiting rooms).

Some microbicide trials have observed trends in decreased adherence over time. Decreased adherence may be due in part to fatigue with clinical trial requirements combined with a product of uncertain benefit. Nonetheless, similar trends may be observed with use of microbicides proven effective, as has been seen with contraceptives. Women's needs for protection will not remain constant over time. A change in partner, or a change in the partner's risk factors, could influence the real and perceived need for continued use of a microbicide, or type of microbicide. Given that women will make their own assessments of the relative risks of sexual behaviours (e.g. concurrent or serial multiple partners), efforts to help women to evaluate and periodically re-evaluate their risks will be needed [41,42].

The clinical requirements of microbicide trial participation have often benefitted the health of the trial participants, but they are also burdensome and complex. Microbicide trials require monthly or quarterly clinic visits that include physical check-ups, collection of blood and urine specimens, gynaecological exams, and sexually transmitted infections and pregnancy testing. Clinical requirements in the context of microbicide introduction are likely to be much less demanding, which could improve continuation.

To date, microbicide trial participants have been counselled to avoid pregnancy since the potential effects of the investigational drug on a foetus are unknown. Increasingly, eligibility criteria for trials include use of an effective contraceptive method and stated willingness to avoid pregnancy for the trial duration. Nevertheless, high pregnancy rates have been observed in many of the microbicide trials [7,43,44]. If refill schedules for effective microbicides are quarterly, or longer, some women using microbicides will become pregnant. Until studies have demonstrated the safety of microbicides for the foetus, contraceptive counselling messages must also be stressed when microbicides are prescribed, and guidance provided on the need to stop product use as soon as pregnancy occurs and contact their health care provider.

### Generalizability of trial participants’ adherence behaviour

To date, more than 20,000 women have participated in large-scale advanced microbicide trials globally (Table 2). Trial participants in the large trials have tended to be in their 20s–30s, with a main partner (e.g. husband) in some trials, or engaged in sex work in others. Participants are typically considered at risk of HIV infection because they reside in high incidence locations, and/or they have multiple sex partners or partners who are migrant workers. Targeting such populations is necessary to determine the safety and efficacy of products in women who are representative of those who could benefit the most from using effective microbicides. However, they may not be representative of those who will actually do so. Being at risk in terms of community prevalence of HIV or risk behaviour patterns may not confer “feeling” or believing oneself to be at heightened risk for HIV [4,45]. Individuals’ self-perception of HIV risk varies within trial populations, and the link between risk perception and consistent product use or retention in trials has not been well established to-date.

Although risk perception as a motivator for adherence in placebo-controlled, blinded clinical trials is not well-known, once an effective product is available, it is highly likely that the women accessing it will perceive themselves to be at risk of HIV. Therefore, it will be important to link supply services to populations at actual high risk by raising awareness of their risk and the availability of microbicides as an HIV prevention strategy. The inclusion of effective microbicides could be a positive new program element in behavioural risk reduction programs. When effective microbicides are made available to women who need them and believe they need them, adherence could be higher than what has been actually achieved in trials.

Some key groups of women have been largely excluded from microbicide efficacy trial participation for ethical and practical reasons, and their sexual and/or health-seeking behaviour may differ in important ways from women who have participated in microbicide trials. These groups include adolescents younger than 16, migrants, older women and pregnant women. There are also women with medical issues that usually preclude trial participation (e.g. latex allergy, genital abnormalities). Women who do not access formal health care services may also have different needs.

## Discussion

It is likely that women's needs for microbicides will be best met if multiple types of products with varying use requirements are available. Some women will prefer a coitally dependent method, some will prefer a continuous-use method, and these needs will change across a woman's sexual life [46–48].

Future adherence to an effective microbicide may exceed the levels achieved in trials since trial participants were repeatedly reminded of the unproven efficacy of products and the randomization to active or placebo arm. Women were also enrolled into trials regardless of their perceived level of personal risk. Alternatively, when the content and frequency of adherence counselling are strained by limited resources typical of service delivery environments, adherence and continuation rates could suffer.

When microbicides are introduced, service delivery programs will focus on achieving uptake and continued use over a sufficient period of time to demonstrate a positive public health impact. Important knowledge has been gained from microbicide clinical trials about factors that are associated with inconsistent use of microbicide products (as well as HIV risk factors). These insights are invaluable for the development of microbicide promotion strategies and targeted counselling for those that need more support.

This article has not focused on clinical trial findings pertaining to microbicide acceptability [49,50]. It is often conjectured that high acceptability supports high adherence, but clinical trial participants are asked to adhere to product use as a study requirement, not because they find the product acceptable. High acceptability also does not necessarily correlate with high willingness to use [51] and, thus, cannot reliably predict future uptake [52]. However, trial participants will probably not be adherent users of a product that they find clearly unacceptable, and those who say they are unwilling to use a product probably will not use it once it is available on the market. Conversely, factors that have been associated with acceptability in clinical trials, such as improved sexual pleasure (for either or both partners) [53,54], could be incorporated into messaging for microbicide introduction.

Many of the adherence issues presented here are not directly associated with the objectives of microbicide clinical trials. Thus, although relevant data may be housed in trial datasets, they may not have been analyzed to investigate the social and behavioural adherence implications raised here since they were not required for primary or secondary analyses. Further, when no reduction in HIV was observed, there was often little incentive to conduct further analyses. These data should be analyzed for potential contributions to better understanding the use factors outlined in this article. Secondary or meta-analyses could provide important information for those developing programs, counselling messages, use-instructions and social marketing campaigns. Clinical trial safety data will inform development of contraindications for use, and advice to future consumers about when to seek medical attention and/or discontinue use.

Women in clinical trials are told “we don't know if it works”, which is a very different message than “we know it works if you use it in this way”. The social and behavioural science data collected within microbicide clinical trials cannot provide clear indicators of behaviour to reliably predict use-behaviour of an approved product in non-trial settings. Nevertheless, a number of trials have adopted “motivational” and “person-centred” counselling to support or improve adherence, and we can expect an increasing body of evidence about the potential for such approaches to strengthen correct, consistent and continued microbicide use. Some of the adherence challenges that trial participants face highlight larger issues of gender inequality. The introduction of effective microbicides, and appropriate counselling on how to use them correctly and consistently, could create inroads for women's empowerment while reducing their risk of HIV infection.
